# Supplementary material for: Altered microRNA Transcriptome in Cultured Human Liver Cells upon Infection with Ebola Virus
Source: Int J Mol Sci. 2021 Apr 6;22(7):3792. doi: 10.3390/ijms22073792 (PMC8038836; doi:10.3390/ijms22073792)
Supplement: Supplementary file 1 [file ijms-22-03792-s001.zip › Supplementary_File/C_ GO_Analysis_Results/16-30nt_go_Mayinga-24h-Huh7_vs_Control-24h-Huh7_down.mature_mirna_targets/GeneOntology_FoldEnrichment.pdf]

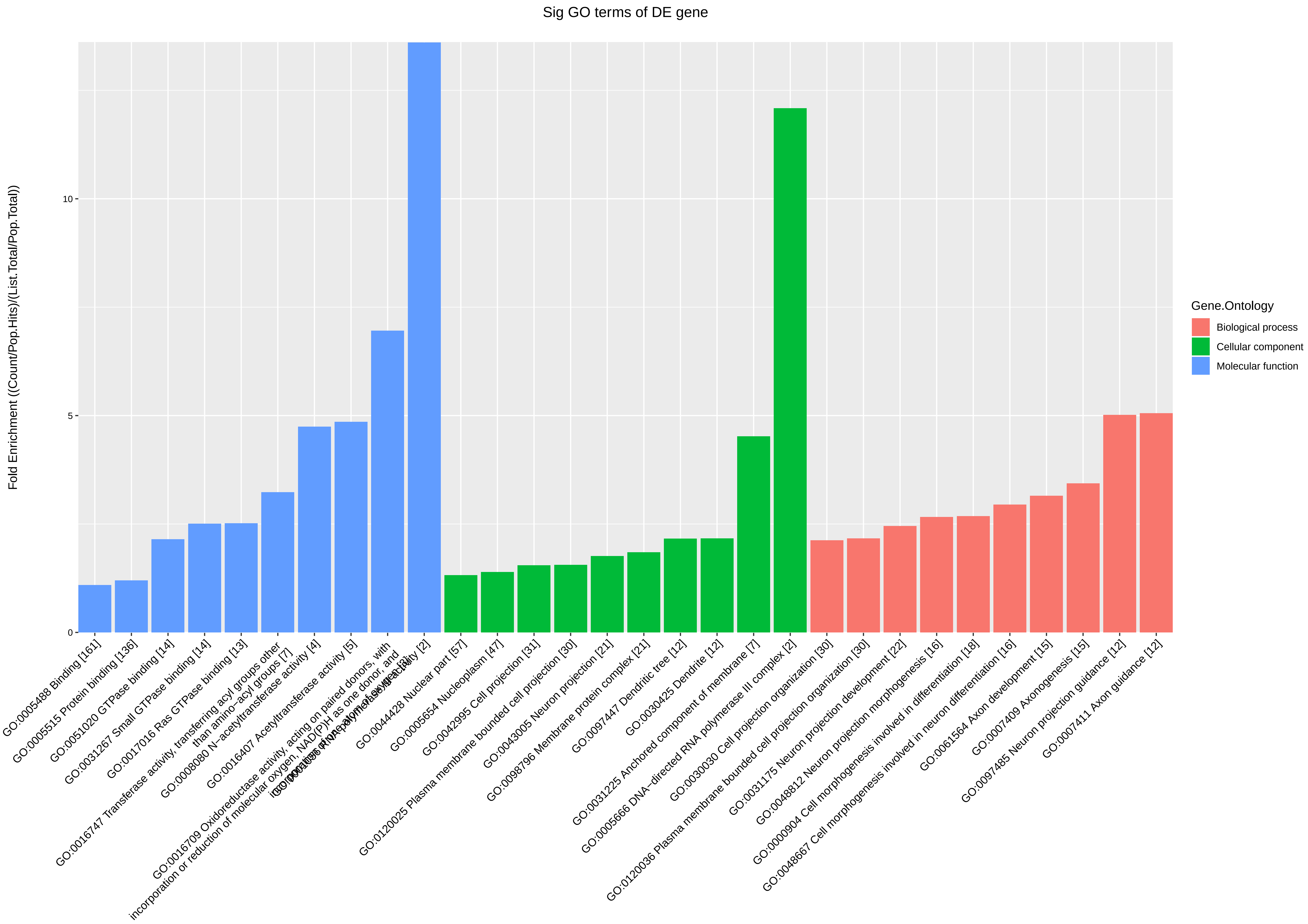
Sig GO terms of DE gene
Fold Enrichment ((Count/Pop.Hits)/(List.Total/Pop.Total))
0
5
10
GO:0005488 Binding [161]
GO:0005515 Protein binding [136]
GO:0051020 GTPase binding [14]
GO:0031267 Small GTPase binding [14]
GO:0017016 Ras GTPase binding [13]
GO:0016747 Transferase activity, transferring acyl groups other than amino−acyl groups [7]
GO:0008080 N−acetyltransferase activity [4]
GO:0016407 Acetyltransferase activity [5]
GO:0016709 Oxidoreductase activity, acting on paired donors, with incorporation or reduction of molecular oxygen, NAD(P)H as one donor, and
GO:0001056 RNA polymerase III activity [2]
GO:0044428 Nuclear part [57]
GO:0005654 Nucleoplasm [47]
GO:0042995 Cell projection [31]
GO:0120025 Plasma membrane bounded cell projection [30]
GO:0043005 Neuron projection [21]
GO:0098796 Membrane protein complex [21]
GO:0097447 Dendritic tree [12]
GO:0030425 Dendrite [12]
GO:0031225 Anchored component of membrane [7]
GO:0005666 DNA−directed RNA polymerase III complex [2]
GO:0030030 Cell projection organization [30]
GO:0120036 Plasma membrane bounded cell projection organization [30]
GO:0031175 Neuron projection development [22]
GO:0048812 Neuron projection morphogenesis [16]
GO:0000904 Cell morphogenesis involved in differentiation [18]
GO:0048667 Cell morphogenesis involved in neuron differentiation [16]
GO:0061564 Axon development [15]
GO:0007409 Axonogenesis [15]
GO:0097485 Neuron projection guidance [12]
GO:0007411 Axon guidance [12]
Gene.Ontology
Biological process
Cellular component
Molecular function
